# COVID-19 vaccine uptake and associated factors among health workers in Kampala city, Uganda

**DOI:** 10.4102/jphia.v16i1.720

**Published:** 2025-02-28

**Authors:** Moses Ocan, Maureen Katusiime, Daniel Kyabayinze, Benon Kwesiga, Rodgers Ayebare, Suzan Nakasendwa, Leah Mbabazi, Henry K. Bosa, Hellen Nansiiro, Daphine Sanger, Mordecai Tayebwa, Boniconsilli Tusiime, Agnes Kiragga, Francis Kakooza, Elizabeth Gonese, Tamrat Shaweno, Nebiyu Dereje, Mosoka P. Fallah, Alex R. Ario

**Affiliations:** 1 Department of Pharmacology and Therapeutics, College of Health Sciences, Makerere University, Kampala, Uganda; 2 Department of Epidemiology, Clinical Services, Ministry of Health, Kampala, Uganda; 3 Department of Public Health, Clinical Services, Ministry of Health, Kampala, Uganda; 4 Department of Epidemiology, Faculty of Public Health, Uganda National Institute of Public Health, Kampala, Uganda; 5 Infectious Diseases Institute, College of Health Sciences, Makerere University, Kampala, Uganda; 6 Clinical Services, Ministry of Health, Kampala, Uganda; 7 College of Health Sciences, Makerere University, Kampala, Uganda; 8 Department of Biostatistics, Faculty of Public Health, African Population and Health Research Center, Kampala, Uganda; 9 Department of Epidemiology, Faculty of Public Health, Africa Centers for Diseases Control and Prevention, Harare, Zimbabwe; 10 Department of Epidemiology, Faculty of Public Health, Africa Centers for Diseases Control and Prevention, Addis Ababa, Ethiopia; 11 Department of Epidemiology, Faculty of Public Health, Africa Centers for Diseases Control and Prevention, Monrovia, Liberia; 12 Department of Epidemiology, Faculty of Public Health, Uganda Institute of Public Health, Kampla, Uganda

**Keywords:** healthcare worker, COVID-19;, vaccine uptake, vaccine hesitancy, health facilities, Uganda

## Abstract

**Background:**

Coronavirus disease 2019 (COVID-19) vaccination is crucial for healthcare workers (HCWs) and understanding their perspectives is vital for promoting vaccine uptake in communities.

**Aim:**

This study assessed factors influencing COVID-19 vaccine uptake among HCWs in Kampala, Uganda.

**Setting:**

A cross-sectional study was done in seven high-volume health facilities in Kampala.

**Methods:**

A questionnaire based on the Behavioural and Social Drivers Model was administered to 200 HCWs. Data were analysed using STATA version 17 to identify associations with vaccination status.

**Results:**

Overall vaccination uptake was 65.2% (95% confidence interval [CI]: 58.1, 71.8), with 17.7% (95% CI: 12.6, 23.7) having received a booster dose. Concerns about safety and side effects were the primary reasons for vaccine hesitancy. While HCWs generally believed that getting vaccinated for diseases such as measles and tuberculosis can help prevent sickness or death, with 70.5% (*n* = 141/200) strongly agreeing, only 48% (*n* = 96/200) of HCWs strongly agreed that vaccination against COVID-19 can reduce the risk of illness or death. Age, profession and facility type were associated with vaccination status.

**Conclusion:**

Healthcare workers in Kampala had high vaccine uptake, but safety concerns remain. Targeted communication strategies from the Ministry of Health are needed to address these concerns and further increase vaccine confidence.

**Contribution:**

This study reveals specific reasons for vaccine hesitancy among HCWs in an African urban setting. These insights inform interventions to improve vaccine uptake in this key population.

## Introduction

The coronavirus disease 2019 (COVID-19) pandemic has undeniably left an indelible mark on global public health, causing widespread loss of life and disrupting livelihoods on an unprecedented scale. The remarkable development and swift evaluation of efficacious vaccines against COVID-19, a triumph of science, provided public health practitioners and policymakers with a potent tool to combat the pandemic, marking a significant shift in our approach to pandemic control.^1^

Despite this remarkable advancement, challenges such as vaccine equity, complex logistics in executing large-scale vaccination campaigns, and lingering scepticism impede the optimal rollout of COVID-19 vaccines.^2^ In Uganda, where the availability of vaccines remains limited, the Ministry of Health (MoH) initially focused on prioritising specific population groups, including frontline healthcare workers (HCWs), the elderly, and individuals with comorbidities.^3^ Later, eligibility expanded to encompass all adults and children aged five and above.^4^

The success of any vaccination programme hinges on a motivated and willing population. Healthcare workers, in particular, play a pivotal role as trusted opinion leaders within their professional circles and the wider community.^5^ Consequently, understanding their perceptions and addressing practical issues related to COVID-19 vaccine uptake presents a significant opportunity for cost-effective strategies to boost vaccine acceptance among HCWs and the vulnerable populations they serve, including the elderly and those with co-morbidities.^6^

As of June 2023, Uganda had administered 26 447 424 COVID-19 vaccine doses, encompassing various vaccines such as Pfizer, Moderna, AstraZeneca and Johnson & Johnson. Of this, 19 207 139 individuals received the first dose, 6 746 228 received the second dose, and 494 057 received a booster dose.^7^ Ahead of opening the economy in January 2022, the MoH also reported that 46% of 22 million priority individuals had been vaccinated, the largest share of which were HCWs.^8^ It is worth observing that the intensified efforts of Uganda’s COVID-19 vaccination programme, managed by the Uganda National Expanded Program on Immunisation (UNEPI), have contributed to the increased vaccine uptake.^9^

However, despite these efforts, the overall COVID-19 vaccine coverage in Uganda, as of June 2022, stood at 62.5% for the general population, falling short of the targets set in the Uganda COVID-19 Vaccination Implementation Plan (UCVIP) to vaccinate all eligible individuals by the end of 2022. Various factors, including insufficient infrastructure, logistical challenges and financial constraints, have contributed to this suboptimal coverage.^2^ Moreover, a study conducted in Uganda by Kabagenyi et al., 2022 identified vaccine hesitancy as a key contributor to low coverage among HCWs, prompting the need to investigate the extent and underlying barriers to vaccine uptake in this group.^10^ Furthermore, the study aimed to identify the most trusted sources of information among HCWs, which could inform targeted risk communication strategies to enhance vaccine acceptance.

Notably, COVID-19 vaccine acceptance rates vary considerably across regions, with Africa being one of the regions reporting lower acceptance rates.^2^ Globally, systematic reviews of vaccine acceptance among HCWs have shown varying rates, ranging from 27.7% in the Democratic Republic of the Congo to 78.1% in Israel.^2^ Previous studies have reported misinformation, fear of side effects and limited knowledge and training as the most common factors associated with the uptake of COVID-19 vaccines among HCWs.^5,6,11,12^ Because HCWs are often the first point of contact with the community, their vaccination status substantially impacts the overall success of nationwide vaccination efforts. In many low- and middle-income countries (LMICs), HCWs are exposed to a high volume of patients because of inadequate staffing, putting them at risk of contracting severe acute respiratory syndrome coronavirus 2 (SARS-CoV-2) and potential vectors for transmission. A study by Ntziora in 2021 reported that unvaccinated HCWs have a 12 times higher risk of SARS-CoV-2 infection.^13^ This underlines the importance of targeting this group for vaccination, both to promote community-wide vaccine uptake and to safeguard the healthcare workforce.^14^

In the light of these critical considerations, understanding and addressing the dynamics of COVID-19 vaccine acceptance among HCWs remains essential in our ongoing battle against the pandemic. This study therefore assessed the COVID-19 vaccine uptake among HCWs in Malawi and the barriers associated with such vaccine uptake.

## Research methods and design

### Study design

We employed a cross-sectional survey among HCWs in Kampala District in Uganda between 21 March 2023 and 31 March 2023.

### Study setting

The study was conducted among HCWs who work in seven high-volume Health facilities in Kampala, which included Mulago National Referral Hospital, Case Hospital, Rubaga Hospital, Makerere University Hospital, Mengo Hospital, Nakasero Hospital and Victoria Hospital. These health facilities were the hotspots for the COVID-19 pandemic in the country.

### Study population and sampling strategy

The study population included physicians, nursing and midwifery personnel, and other HCWs such as radiographers. According to the World Health Organization (WHO), a health worker is a person who provides preventive, curative, rehabilitative and promotional health services based on an extensive body of theoretical and factual knowledge in the diagnosis and treatment of disease and other health problems.^15^

A sample of 200 HCWs was purposively selected to represent the various cadres of health workers in the system that included physicians, nursing and midwifery, pharmaceutical personnel, laboratory HCWs, community support and public health workers, and other health workers otherwise not defined. The sample size was adequately powered to detect the prevalence of vaccine uptake, estimated between 30% and 40% among HCWs in Africa.

The study used purposive sampling of HCWs for participation in the study. A list of HCWs in service was obtained from the health facilities and served as the sampling frame. The research team contacted HCWs willing to participate in the survey and requested them to respond to the questionnaire. Participants whose vaccination status was unknown were excluded.

### Data collection

The data collection tool was designed in REDCap, pretested and revisions made before the study implementation. The research assistants obtained written informed consent. Survey questions based on the Behavioural and Social Drivers model for creating vaccine demand were developed, piloted, and adapted on the REDCap platform. Following consent, the research assistants administered a structured questionnaire to assess HCWs’ uptake, willingness, attitudes and barriers towards COVID-19 vaccines. All data were directly entered into Case Report Forms (CRFs) in REDCap software using mobile gadgets.

The study variables included participants’ demographics, vaccination status, confidence in COVID-19 vaccines reasons why HCWs wouldn’t want to get vaccinated, factors that would enable HCWs that haven’t been boosted or fully vaccinated to complete the vaccine schedule; preferences for the ideal place to get vaccinated and commonly used sources of information about vaccines and level of trust in the stated sources.

Data entry was performed in real-time to support data completeness and accuracy. The research assistants checked data for errors and accuracy before uploading it into the REDCap database. The data managers conducted data cleaning and quality control and generated query reports that the research team resolved. Data were protected by ensuring only the research teams accessed REDCap via password-protected accounts.

### Data analysis

Data were analysed using STATA version 17. Descriptive statistics were obtained as frequencies and percentages for categorical variables. Age was assessed for normality using the Shapiro–Wilk test. Median age and the corresponding interquartile range (IQR) were reported. The difference in the median age between vaccination status in the different groups was tested using the Mann–Whitney test. Comparisons between participant’s categorical characteristics and vaccination status were performed using the Chi-square tests.

The logistic regression model was used to identify the participants’ demographic and work-related characteristics of being unvaccinated against COVID-19 among HCWs. Odds ratios (OR) with their corresponding 95% confidence intervals (CI) were reported. Statistical significance was declared at *p* ≤ 0.25 at bivariate analysis and *p* ≤ 0.05 at multivariable analysis. Confounding was assessed and declared by a 10% percentage increase in the odds ratios in the multivariable model.

### Ethical considerations

The study obtained approval from the School of Biomedical Sciences Research and Ethics Committee (number SBS-2022-261) and the Uganda National Council for Science and Technology (number HS2664ES). After that, clearance was received from the Director General Health Services (DGHS) and administrative clearances from the seven study hospitals. A written informed consent was obtained from participants prior to data collection.

## Results

### Socio-demographic characteristics of study participants

Over half, 51% (*n* = 102/200) of the study participants were female health workers. Most (28.5%, *n* = 57/200) of the respondents were nursing and midwifery healthcare personnel. Most participants, 26% (*n* = 53/200), were aged 28–32 years with a median (interquartile range [IQR]) age of 34 (29, 41) years. Most respondents, 43% (*n* = 86/200) worked in public health facilities (Table 1).

**TABLE 1:** Demographics characteristics of healthcare workers (*N* = 200) in healthcare facilities in Kampala city, March 2023.

Characteristics	Description	Frequency (*n*)	%	Median	IQR
Age in years	-	-	-	34	29, 41
Age categories (years)	23–27	35	17.5	-	-
	28–32	53	26.5	-	-
	33–37	44	22.0	-	-
	38–42	21	10.5	-	-
	43–47	18	9.0	-	-
	48 >	28	14.0	-	-
	*Missing*	*1*	*0.5* *	-	-
Gender	Female	102	51.0	-	-
	Male	95	47.5	-	-
	*Missing*	*3*	1.5*	-	-
Healthcare worker category	Physician	45	22.5	-	-
	Nursing and midwifery	57	28.5	-	-
	Pharmaceutical personnel	19	9.5	-	-
	Laboratory health worker	36	18.0	-	-
	Community support and public health worker	21	10.5	-	-
	Other health workers	20	10.0	-	-
	*Missing*	*2*	1.0*	-	-
Healthcare facility	Mulago National Referral Hospital	50	25.0	-	-
	Case Hospital	25	12.5	-	-
	Lubaga Hospital	24	12.0	-	-
	Makerere University Hospital	25	12.5	-	-
	Mengo Hospital	25	12.5	-	-
	Nakasero Hospital	25	12.5	-	-
	Victoria Hospital	25	12.5	-	-
	*Missing*	*1*	0.5*	-	-
Health facility sector	Public	86	43.0	-	-
	Private	64	32.0	-	-
	Private not-for-profit	47	23.5	-	-
	*Missing*	*3*	1.5*	-	-

IQR, interquartile range.

* , Missing observations. *P*-values were obtained for complete data.

### The proportion of vaccinated healthcare workers among study participants

**FIGURE 1:**
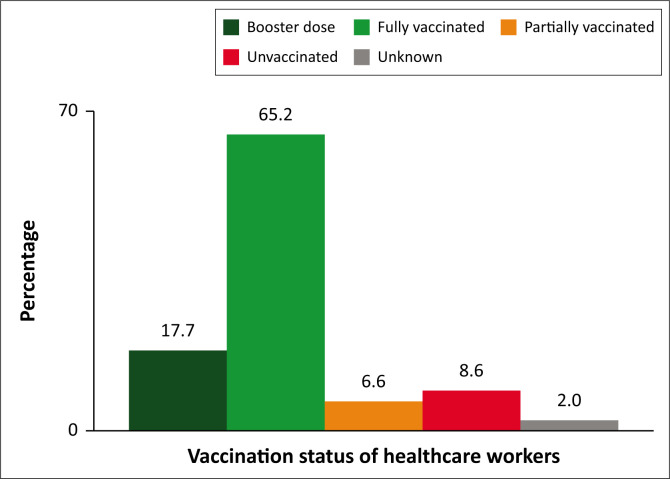
Showing status of COVID-19 vaccination among healthcare workers.

The majority, 88.5% (95% CI: 83.2, 92.6) of HCWs had received at least one dose of COVID-19 vaccine. Most HCWs were fully vaccinated, 65.2% (95% CI: 58.1, 71.8). In addition, one in every six healthcare personnel had received a booster dose of COVID-19 vaccine. Some, 8.6% (95% CI: 5.0, 13.3) of the HCWs had not received any COVID-19 vaccine (Figure 1).

### Comparison of vaccinated and unvaccinated healthcare workers

Of the unvaccinated HCWs, the majority, 64.7% (*n* = 11/17) were females. Over one in three unvaccinated HCWs are physicians. There was no statistically significant association (*p* > 0.05) between vaccination status and demographic characteristics (Table 2).

**TABLE 2:** Comparison of the coronavirus disease 2019 vaccinated and unvaccinated healthcare workers (*N* = 200) in healthcare facilities in Kampala city, March 2023.

Characteristics	Description	Unvaccinated (*n* = 17)				Vaccinated (*n* = 177)				*P*
		*n*	%	Median	IQR	*n*	%	Median	IQR	
Age in years		-	-	30	30, 34	-	-	29	34, 42	0.251
Age category (years)	23–27	2	11.8	-	-	33	18.6	-	-	0.271
	28–32	9	52.9	-	-	43	24.3	-	-	-
	33–37	2	11.8	-	-	40	22.6	-	-	-
	38–42	1	5.9	-	-	19	10.7	-	-	-
	43–47	2	11.8	-	-	16	9.0	-	-	-
	48 and above	1	5.9	-	-	26	14.7	-	-	-
Gender	Female	11	64.7	-	-	91	51.4	-	-	0.322
	Male	6	35.3	-	-	86	48.6	-	-	-
Healthcare worker cadre	Physician	6	35.3	-	-	37	20.9	-	-	0.141
	Nursing and midwifery	3	17.7	-	-	52	29.4	-	-	-
	Pharmaceutical personnel	4	23.5	-	-	14	7.9	-	-	-
	Laboratory health worker	3	17.7	-	-	33	18.6	-	-	-
	Community support and public health worker	0	0.0	-	-	21	11.9	-	-	-
	Other health workers	1	5.9	-	-	19	10.7	-	-	-
	*Missing*	*0*	*0.0*	-	-	*1*	0.6 *	-	-	-
Health facility level	National referral hospital	7	41.2	-	-	41	23.2	-	-	0.122
	General Hospital	10	58.8	-	-	-	-	-	-	-
	Health Centre IV	0	0.0	-	-	1	0.6	-	-	-
	*Missing*	*0*	0.0*	-	-	*1*	0.6 *	-	-	-
Health facility sector	Public	9	52.9	-	-	74	41.8	-	-	0.497
	Private	6	35.3	-	-	57	32.2	-	-	-
	Private not-for-profit	2	11.8	-	-	45	25.4	-	-	-
	*Missing*	*0*	0.0 *	-	-	*1*	0.6 *	-	-	-

IQR, interquartile range.

* , Missing observations. *P*-values were obtained for complete data.

Among vaccinated HCWs with at least one dose, 73.1% (*n* = 128/175) found it easy to get vaccination services alone.

Over a third, 48% of HCWs had their complete trust in information about COVID-19 and vaccination provided by the MoH, followed by international television (26.8%), local television (25.9%) and other health workers (25.6%). Some HCWs, 25.3% did not trust the information provided on social media. The presentation of HCW responses in alignment with the Behavioral and Social Drivers of Vaccination (BeSD) framework can be accessed in Table 1.

The majority, 83.5% (*n* = 167/200) of the HCWs preferred getting their COVID-19 vaccines in a hospital. Other HCWs preferred getting their COVID-19 vaccine from health centres/clinics (18%, *n* = 36/200), community centres (meeting centres, halls, and local shops) (6.5%, *n* = 13/200), pharmacies (2.5%, *n* = 5/200) and churches/homes (2.5%, *n* = 5/200).

More than half, 63.5% (*n* = 125/197) of the HCWs would recommend COVID-19 vaccination to other community members. Most, 46.7% (*n* = 92/197) of the HCWs would recommend the booster vaccine doses the Uganda MoH recommended.

### Reasons for not being fully vaccinated/or boosted among healthcare workers

There were several reasons for low vaccine uptake among HCWs who were not fully vaccinated. Vaccine safety and fear of side effects were the most frequent reasons why some HCWs were not yet fully vaccinated and/or boosted (Table 3).

**TABLE 3:** Reasons why the healthcare workers are not yet fully vaccinated and/or boosted.

Number	Multiple reasons	Frequency (*n*) (*n* = 34)	%
1	I am concerned about the safety of the vaccine, including side effects	11	32.4
2	No interest, fear, pregnancy and strong immunity	6	17.7
3	I do not wish to respond	4	11.8
4	I am waiting to see how the vaccine affects other people that I know	4	11.8
5	Waiting for the window of eligibility to open	2	5.9
6	I do not have time to get vaccinated	2	5.9
7	I do not want to miss work	1	2.9

Among the reasons for not being vaccinated, 28.6% of HCWs felt the development and/or authorisation of the vaccine was rushed, and it may not be thoroughly tested, 23.8% were concerned about serious side effects, and 19.0% of HCWs had already been infected with COVID-19 and were not worried about being re-infected.

The HCWs reported the following as reasons for them not getting booster COVID-19 vaccine: need for more information on vaccine safety and efficacy (55.9%), full approval of vaccine from regulatory authorities (23.5%), nothing and will not get vaccinated (23.5%).

### Factors associated with COVID-19 un-vaccination among healthcare workers in selected health facilities in Kampala city, Uganda

Healthcare workers aged 48 years and above were less likely to be unvaccinated than those aged 23–27 years (adjusted odds ratio [aOR] = 0.73, 95% CI: 0.05, 10.5). Pharmaceutical personnel were twice more likely to be unvaccinated than physicians (aOR = 2.19, 95% CI: 0.41, 11.5). The HCWs in general hospitals were less likely to be unvaccinated than those in National referral hospitals (aOR = 0.87, 95% CI: 0.10, 7.73). However, all associations were not statistically significant (Table 4).

**TABLE 4:** Logistic regression model to assess the association of demographic characteristics and coronavirus disease 2019 unvaccination among healthcare workers in Kampala city, March 2023.

Characteristic	Crude OR	95% CI	Adjusted OR	95% CI	Adjusted *p*
**Age in years**					
23–27	Ref.	Ref.	Ref.	Ref.	-
28–32	3.45	0.69, 17.07	6.01	0.94, 38.20	0.058*
33–37	0.83	0.11, 6.18	1.19	0.13, 10.63	0.878
38–42	0.87	0.07, 10.2	1.36	0.97, 19.00	0.819
43–47	2.06	0.27, 16.00	2.59	0.27, 24.82	0.409
48 >	0.63	0.05, 7.39	0.73	0.05, 10.53	0.816
**Gender**					
Female	Ref.	Ref.	Ref.	Ref.	-
Male	0.58	0.20, 1.63	0.43	0.13, 1.41	0.165
**Cadre**					
Physician	Ref.	Ref.	Ref.	Ref.	-
Nursing and midwifery	0.36	0.08, 1.51	0.29	0.05, 1.56	0.215
Pharmaceutical personnel	1.76	0.43, 7.19	2.19	0.41, 11.5	0.312
Laboratory health worker	0.56	0.13, 2.42	0.61	0.12, 3.11	0.549
Other health workers	0.32	0.04, 2.89	0.35	0.04, 3.48	0.376
**Health centre structure**					
National Referral Hospital	Ref.	Ref.	Ref.	Ref.	-
General Hospital	0.53	0.19, 1.48	0.87	0.10, 7.73	0.902
**Health facility sector**					
Public	Ref.	Ref.	Ref.	Ref.	-
Private	0.87	0.29, 2.57	0.53	0.06, 4.32	0.551
Private not-for-profit	0.37	0.08, 1.77	0.27	0.02, 2.95	0.280

aOR, adjusted odds ratio; Ref., reference category; CI, confidence intervals.

* , Borderline at 0.05 < *p* < 0.06.

### Trust in the sources of information regarding COVID-19 vaccines

In this survey on trust in information sources, the MoH emerges as the most trusted source, with 48.0% of the respondents expressing complete trust in it. The other most trusted sources of information included local television (25.9%) and international television (26.8%). Moreover, friends, family, and local radio have notable levels of trust, with 51.5% and 43.8% of respondents having at least a little trust in them, respectively.

On the other hand, social media appeared to be the least trusted information source, with only 10.6% expressing complete trust and 25.3% indicating no trust at all. Local religious leaders and supervisors at work also rank among the least trusted sources, with 13.8% and 7.7% of respondents expressing no trust in them, respectively (Table 5).

**TABLE 5:** Trusted sources of information for the healthcare workers on coronavirus disease 2019 vaccine.

Information source	Level of trust									
	Completely		Mostly		A little		Not at all		Do not know/Did not get information	
	*n*	%	*n*	%	*n*	%	*n*	%	*n*	%
Local television (*n* = 197)	51	25.9	51	25.9	72	36.6	17	8.6	6	3.1
International television (*n* = 198)	53	26.8	53	26.8	62	31.3	21	10.6	9	4.6
Local radio (*n* = 194)	38	19.6	44	22.7	85	43.8	21	10.8	6	3.1
Social media (*n* = 198)	21	10.6	23	11.6	99	50.0	50	25.3	5	2.5
Website (*n* = 198)	40	20.2	60	30.3	74	37.4	16	8.1	8	4.0
Newspapers (*n* = 195)	27	13.9	65	33.3	83	42.6	13	6.7	7	3.6
Magazines (*n* = 197)	19	9.6	31	15.7	105	53.3	28	14.2	14	7.1
Friends and family (*n* = 196)	21	10.7	40	20.4	101	51.5	31	15.8	3	1.5
Ministry of Health (*n* = 196)	94	48.0	66	33.7	23	11.7	3	1.5	10	5.1
Other health workers (*n* = 195)	50	25.6	88	45.1	44	22.6	7	3.6	6	3.1
Community leaders (*n* = 195)	25	12.8	43	22.1	95	13.3	26	13.3	6	3.1
Local religious leaders (*n* = 196)	34	17.3	38	19.4	94	48.0	27	13.8	3	1.5
Boss/supervisor (*n* = 194)	39	20.1	70	36.1	63	32.5	15	7.7	7	3.6

## Discussion

The study found that most HCWs trusted COVID-19 vaccine information provided by the MoH compared to that from the WHO. The findings of a similar study by Ouni et al. among health workers in northern Uganda showed that most health workers trusted information from the MoH.^12^ The high level of trust in COVID-19 vaccine information provided by the MoH, compared to that of the WHO, indicates the importance of collaboration in science communication. Local ministries of health are crucial in advocating for vaccine uptake, especially among HCWs in LMICs. There is a need for the WHO and other development partners to work through the local ministries of health to communicate information on health innovations to the local population.

The most mentioned reason for HCWs not taking the COVID-19 vaccine included concerns that the vaccine development and/or authorisation by the National Drug Regulatory Agency was rushed. In addition, HCWs reported concerns regarding COVID-19 vaccine safety and side effects as a reason for not being vaccinated. This is like the findings of previous studies that reported concerns by HCWs in vaccine development, lack of long-lasting immunity, safety, and side effects as barriers to COVID-19 vaccine uptake.^16,17^ The development of the COVID-19 vaccine used advanced mRNA technology that most of the scientific community, especially in low-income countries, were unfamiliar with. In addition, the detection and reporting of adverse reactions associated with the COVID-19 vaccine in the early days of its administration and the shortened development period potentially affected vaccine uptake globally, especially among HCWs. There is a need for the MoH to explore further in order to establish key concerns by HCWs and develop strategies to help improve the uptake of the COVID-19 vaccine and other vaccines in the country.

Our research indicates that the majority of HCWs in hospitals in Kampala city have been fully vaccinated and would also advise members of the community to get the COVID-19 vaccine. While many HCWs are highly confident in the COVID-19 vaccines, it was also observed that they believe that having more information on vaccine safety and effectiveness, as well as full approval of the vaccine from regulatory agencies, would help them make better decisions regarding vaccination or getting a booster shot. The high prevalence of COVID-19 vaccinated HCWs is similar to findings of a study by Ouni et al., 2023, which reported a low vaccine hesitancy among HCWs.^12^ Our findings are also concur with those of a previous review study by Sallam, 2020, which reported high COVID-19 vaccine acceptance among healthcare ranging from 27.7% to 78.1%.^2^ The significant uptake of vaccines among HCWs could be a result of focused advocacy and promotional efforts by the MoH, the WHO and the Centers for Disease Control and Prevention (CDC), all of which have been encouraging vaccination for healthcare professionals.^18^ However, the scarcity and inconsistency of information regarding the safety and side effects of COVID-19 vaccines have impeded wider acceptance of the vaccine among the population. Specifically, HCWs have shown reluctance towards receiving the COVID-19 vaccine, largely because of a lack of detailed information concerning its registration and official approval by local regulatory bodies. Addressing this issue is critical for the success of future vaccine distribution efforts, underscoring the need for making trustworthy and easily obtainable information on vaccine safety and side effects available.

The study showed that most of the HCWs had knowledge of where to get the COVID-19 vaccine and reported finding it easy to access the vaccine. The majority would prefer getting their COVID-19 vaccine in a hospital. The behavioural and social determinants of vaccine uptake are essential in vaccine rollout programmes. The ease of access to the COVID-19 vaccine reported among HCWs in this study potentially contributed to the high vaccine uptake among HCWs. Because vaccines were primarily administered in health facilities, it was thus more accessible for HCWs to access the COVID-19 vaccines; however, in most low-income countries, access to health facilities by the general population during the pandemic was low and could potentially present a barrier to vaccine uptake.

The study examined the factors influencing COVID-19 vaccine acceptance among HCWs in high-volume hospitals in Kampala city. It was found that HCWs aged 28–32 years were less likely to receive the COVID-19 vaccine compared to those aged 23–27 years. Furthermore, HCWs at Makerere University Hospital were more inclined to have been vaccinated against COVID-19 than those at Mulago National Referral Hospital. These findings are similar to those of a previous study conducted by Mudhune et al. in 2023.^19^ The WHO and CDC prioritised HCWs to receive the COVID-19 vaccine.^18,20^ This is because the high risk of exposure to SARS-CoV-2 virus among HCWs. A study by Nguyen et al., 2020 found that HCWs are three times more at risk of getting COVID-19 than the general population.^21^ Gaining buy-in from doctors and nurses is vital for greater public support for vaccines, as patients demonstrate high trust in vaccinators. In this study, HCWs aged 28–32 years and those working in a university hospital (general hospital) were likely to get the COVID-19 vaccine. This could be because of the difference in risk perception among HCWs aged 28–32 years and those working in a university hospital from the rest.^22^ Furthermore, HCWs in university hospitals are potentially exposed to quality evidence and have a better perception of vaccine efficacy, side effects, and safety, which are critical barriers to COVID-19 vaccine uptake. The high uptake of the COVID-19 vaccine among HCWs serving in a university hospital (Health Center IV) could be because of the knowledge of COVID-19 disease and the vaccine, mainly because of ongoing research studies at the facility.

Regarding trusted sources of information, the findings suggest that individuals place a significant amount of trust in information about COVID-19 vaccines obtained from local and national health authorities and in television broadcasts. This finding is in contrast to what was found in a study by Fegus et al. (2023), where social media and peers were the most used and trusted sources of information.^23^ The reduced trust in social media and supervisors/peers indicates a paradigm shift with traditional media and local authoritative bodies re-establishing trust and newer digital platforms and personal connections falling out of favour.

The study had some limitations, such as recall bias of the HCW vaccination status, which we could not verify in this survey. However, because data collection was performed during campaigns for vaccination in the general public and specifically among the at-risk population, this potentially reduced the effect of recall bias on the study’s findings. Furthermore, the purposive sampling approach used for this study means our results cannot be generalisable to Uganda’s HCW population.

## Conclusion

There was high uptake of the COVID-19 vaccine among HCWs in health facilities in Kampala city, with most respondents reporting easy access to vaccinations. Although HCWs would recommend the vaccine to their community, many expressed concerns regarding its safety and efficacy. To address this, it is essential for the MoH and other risk communication stakeholders to continually engage with HCWs on the approval process and safety of available vaccines.
